# Silymarin suppresses hepatic stellate cell activation in a dietary rat model of non-alcoholic steatohepatitis: Analysis of isolated hepatic stellate cells

**DOI:** 10.3892/ijmm.2012.1029

**Published:** 2012-06-14

**Authors:** MINA KIM, SU-GEUN YANG, JOON MI KIM, JIN-WOO LEE, YOUNG SOO KIM, JUNG IL LEE

**Affiliations:** 1 Department of Internal Medicine, Division of Gastroenterology, Inha University School of Medicine, Jung-Gu, Incheon;; 2 Utah-Inha DDS and Advanced Therapeutics Research Center, Yeonsu-Gu, Incheon;; 3 Department of Pathology, Inha University School of Medicine, Jung-Gu, Incheon, Republic of Korea

**Keywords:** non-alcoholic steatohepatitis, methionine- and choline-deficient diet, insulin resistance, hepatic stellate cell, silymarin

## Abstract

Non-alcoholic steatohepatitis (NASH) is characterized by hepatocellular injury and initial fibrosis severity has been suggested as an important prognostic factor of NASH. Silymarin was reported to improve carbon tetrachloride-induced liver fibrosis and reduce the activation of hepatic stellate cells (HSC). We investigated whether silymarin could suppress the activation of HSCs in NASH induced by methionine- and choline-deficient (MCD) diet fed to insulin-resistant rats. NASH was induced by feeding MCD diet to obese diabetic Otsuka Long-Evans Tokushima Fatty (OLETF) rats. Non-diabetic Long-Evans Tokushima Otsuka (LETO) rats were fed with standard chow and served as the control. OLETF rats were fed on either standard laboratory chow, or MCD diet or MCD diet mixed with silymarin. Histological analysis of the liver showed improved non-alcoholic fatty liver disease (NAFLD) activity score in silymarin-fed MCD-induced NASH. Silymarin reduced the activation of HSCs, evaluated by counting α-smooth muscle actin (SMA)-positive cells and measuring α-SMA mRNA expression in the liver lysates as well as in HSCs isolated from the experimental animals. Although silymarin decreased α_1_-procollagen mRNA expression in isolated HSCs, the anti-fibrogenic effect of silymarin was not prominent so as to show significant difference under histological analysis. Silymarin increased the nuclear translocation of nuclear factor erythroid 2-related factor 2 (Nrf2) and decreased tumor necrosis factor (TNF)-α mRNA expression in the liver. Our study suggested that the possible protective effect of silymarin in diet induced NASH by suppressing the activation of HSCs and disturbing the role of the inflammatory cytokine TNF-α.

## Introduction

Non-alcoholic steatohepatitis (NASH) is a progressive form of non-alcoholic fatty liver disease (NAFLD). Unlike simple steatosis, NASH is characterized by hepatocellular injury accompanied by inflammation and fibrosis (1). Although factors that might forecast the progression of NAFLD have not been clearly established, many studies have suggested that initial fibrosis severity might be one of the most important prognostic factors (2–4). Therefore, preventing and treating liver fibrosis might be an optimal treatment goal for NAFLD. NAFLD is generally included as a component of the metabolic syndrome where insulin resistance plays a critical role (5–7). A rat model of NASH with insulin resistance has been reported, by feeding a methionine- and choline-deficient (MCD) diet to an established animal model of obese type 2 diabetes (8,9). Although MCD diet alone was reported to be sufficient to induce severe steatosis and necroinflammation by generating oxidative stress (10), feeding MCD diet to rats with generalized insulin resistance accelerated NASH (8). Using this animal model it has been reported that administration of an antifibrogenic agent improved NASH (9).

Silymarin, a mixture of flavonoliganans extracted from the milk thistle (*Silibum marianum*), has demonstrated the protective effects in hepatocytes exposed to various chemicals and toxins (11–14). The mechanism of this protective effect has not been delineated, although it is often explained by silymarin action as an antioxidant (15). Silymarin is also reported to ameliorate carbon tetrachloride (CCl_4_) induced liver fibrosis and reduced activation of hepatic stellate cells (HSCs) (16).

We hypothesized that silymarin would suppress the activation of HSCs in MCD diet fed insulin resistant rats, thereby ameliorating NASH. In order to test this hypothesis, silymarin was concomitantly administered with MCD diet to insulin-resistant rats, and an *ex vivo* study on HSCs performed by isolating HSCs from these rats.

## Materials and methods

### Materials

Silymarin was purchased from Sigma Chemicals Co. (St. Louis, MO, USA). The MCD diet was obtained from Research Diets, Inc. (New Brunswick, NJ, USA).

### Animals and treatment

Male Otsuka Long-Evans Tokushima Fatty (OLETF) rats, an established animal model of obese type 2 diabetes (8), were used (Otsuka Pharmaceutical, Tokushima, Japan). OLETF rats were reported to show obesity and hyperinsulinemia from 8 weeks of age, and demonstrated the hepatic accumulation of fat in an age-dependent manner (17,18). As the control animals, Long-Evans Tokushima Otsuka (LETO) rats, which originated from the same colony as the OLETF rats by selective mating and did not develop diabetes, were used. Both OLETF and LETO rats were 4-weeks-old and were housed in a room under controlled temperature (23°C), humidity, and lighting (12-h artificial light and dark cycle). Animals were given free access to the standard laboratory rat chow and tap water. At 24 weeks of age, rats were divided into experimental groups and fed for 8 weeks. OLETF rats were fed on one of three different diets as follows: the standard laboratory rat chow (OLETF/vehicle, n=10), the MCD diet (OLETF/MCD, n=10), and the MCD diet mixed with silymarin (OLETF/MCD+silymarin, silymarin content 0.5% w/w, n=10). LETO rats were continued to be fed on the standard laboratory rat chow (LETO/vehicle, n=10). Although all rats were allowed unrestricted access to water, OLETF and LETO rats, fed on the standard chow were pair-fed with either the MCD or with MCD and silymarin. The body weight and food intake in each group of rats were recorded every week. All animal procedures were performed in accordance with the guidelines set by the Institutional Animal Care and Use Committee at Inha University School of Medicine. After 8 weeks of feeding on experimental diets, rats were sacrificed.

### Immunohistological analysis

Sections of liver tissue specimens, fixed in 10% formalin and embedded in paraffin wax, were stained with H&E and Masson’s trichrome for histological examination. A blinded investigator (J.M.K.) evaluated the slides for fatty change, inflammation existence of hepatocyte ballooning and fibrosis as described in previous studies with minor modifications (19–22). The degree of steatosis was scored as the percentage of hepatocytes containing macrovesicular fat (grade 0, no steatosis; grade 1, <25%; grade 2, 26–50%; grade 3, 51–75%; grade 4, 76–100%). Inflammation was histologically quantified by counting inflammatory foci in 20 consecutive high-power fields (40x objective) (average histological grade, grade 0, no foci; grade 1, <2 foci per high-power field; grade 2, ≥2 foci per high-power field). The individual scores of steatosis, inflammation and hepatocyte ballooning were added to produce an overall score, namely NAFLD activity score (NAS) as previously suggested (21). Fibrosis scores were as follows: 1, pericellular and perivenular fibrosis; 2, focal bridging fibrosis; 3, bridging fibrosis with lobular distortion; and 4, cirrhosis.

Sections of liver tissue specimens were immunostained with mouse anti-human α-smooth muscle actin (α-SMA) (Dako, Carpinteria, CA, USA). Detection of the primary antibody was carried out by immunoperoxidase technique using the ABC kit (Vector Laboratories). Peroxidase activity was identified by reaction with diaminobenzidine tetrahydrochloride substrate (DAB). Data are represented as the number of α-SMA positive cells present in thirty 40x fields (1.3 mm^2^, approximately 3,000 hepatocytes).

### Hepatic stellate cell isolation

It has been previously reported that HSCs could be isolated from the injured liver using a conventional density gradient centrifugation method (23). In this study, HSCs were isolated from the livers of each experimental group by in situ perfusion using collagenase and pronase (24). The viability and purity of HSC preparations were consistently found to be >95% as accessed via trypan blue (Gibco-BRL, Grand Island, NY, USA) exclusion and autofluorescence, respectively (25,26).

### RNA extraction and real-time polymerase chain reaction (PCR)

Total-RNA was extracted from either frozen whole liver or isolated HSCs using TRIzol reagent (Invitrogen, Carlsbad, CA, USA) according to the manufacturer’s protocol. RNA samples were quantified by spectrophotometry. The RNA integrity was assessed using agarose gel electrophoresis and ethidium bromide staining. The RNA samples were then diluted in RNase-free water and stored at −70°C until use.

RNA (5 μg) were reverse-transcribed using the RNA PCR kit version 1.2 (Takara Bio, Inc., Japan) according to the manufacturer’s recommendations. Oligonucleotide primers and the TaqMan probe for α_1_-procollagen, α-SMA, sterol regulatory element-binding protein-1c (SREBP-1c), tumor necrosis factor-α (TNF-α) and glyceraldehyde-3-phosphate dehydrogenase (GAPDH) internal control were obtained from Perkin-Elmer Applied Biosystems (Foster City, CA, USA), purchased as a ready-for-use form in Assays-on-Demand Gene Expression products. The TaqMan probe was labeled at the 5′ end with the reporter dye FAM and at the 3′ end with the quencher TAMRA. PCR was carried out in triplicate for each sample on a Bio-Rad iCycler Optical Module (Bio-Rad Laboratories, Inc., Hercules, CA, USA). Each 20-μl reaction contained 10 μl of TaqMan Universal PCR Master Mix, 1 μl of Assays-on-Demand Gene Expression Assay Mix and 9 μl of cDNA diluted in RNase-free water. All reactions were carried out using the following cycling parameters: 95°C for 10 min, followed by 40 cycles of 95°C for 15 sec and 60°C for 1 min. mRNA fold changes in target genes relative to the endogenous GAPDH control were calculated as suggested in previous studies (27).

### Preparation of cytosolic and nuclear fractions

The cytosolic and nuclear fraction of the protein was extracted from liver tissue using a protein extraction kit (Bio-Rad Laboratories, Inc.) according to the manufacturer’s instruction. Briefly, 50 mg of liver tissue was added into the chilled the Dounce homogenizer with 0.75 ml of cytoplasmic protein extraction buffer (CPEP) and was broken up by stroking. After incubating Dounce homogenizer containing the homogenate on ice for 2 min, the supernatant was transferred using a pipette. The cell lysate was centrifuged at 1,000 x g for 10 min at 4°C. Upon completion of the centrifugation, the supernatant containing cytoplasmic protein was transferred and centrifuged, and the pellet containing nuclei was washed using CPEP. The protein concentration was determined with a Lowry protein assay (Bio-Rad Laboratories, Inc.).

### Western blotting

Whole cell extracts were prepared from HSCs which were treated as described above by using Triton lysis buffer containing protease and phosphatase inhibitors as described else where (9). Total hepatic or whole cell extract protein was estimated using bovine serum albumin as a standard (DC protein assay; Bio-Rad Laboratories, Inc.).

Whole cell protein (50 μg) or cytosolic/nuclear protein (50 μg) was separated by 10% sodium dodecyl sulfatepolyacrylamide gel electrophoresis and the resolved proteins were transferred to a nitrocellulose membrane (Schleicher and Schuell, Middlesex, UK). The membrane was blocked with 5% skim milk in 10 mM Tris-HCl containing 150 mM NaCl and 0.5% Tween-20 [Tris-buffered saline (TBS)-T]. After washing with TBS-T, the membrane was then incubated with 1:1,000 dilution of specific primary antibodies against phospho-extracellular signal-related protein kinase (ERK) 1/2 for whole cell protein and nuclear factor erythroid 2-related factor 2 (Nrf2) (Santa Cruz Biotechnology Inc., Santa Cruz, CA, USA) for the cytosolic/nuclear protein. The membrane was washed and then incubated with a horseradish peroxidase (HRP)-conjugated secondary antibody (New England Biolabs, Beverly, MA, USA) diluted 1:2,000. After washing with TBS-T, the membrane was developed using an enhanced chemiluminescence detection kit (Amersham, Piscataway, NJ, USA). Anti β-actin Ab (Cell Signaling Technology, Inc., Danvers, MA, USA) was used to verify the equal loading of the protein samples.

### Statistical analyses

All results are expressed as means or means ± standard deviation of the mean (SD). Data were analyzed by nonparametric analysis (Kruskal-Wallis or Mann-Whitney U test) and P<0.05 was considered statistically significant. All calculations were performed with SPSS version 12.0 software for Windows (SPSS Inc., Chicago, IL, USA).

## Results

### Body and liver weight of the rat

The initial weight of LETO rats, non-diabetic control animals for OLETF, was significantly lower as expected. The MCD diet-fed rats had lower final body weight compared to the standard chow-fed rat groups after 8 weeks despite the pair feeding as previously reported (8,28,29). However, the general condition of the MCD-fed rats remained healthy. Relative liver weight (liver weight/final body weight) of the MCD-fed rats was significantly higher compared to that of the standard chow-fed rats. Adding silymarin failed to reverse the increased relative liver weight (Table I).

### Silymarin enhances translocation of Nrf2 protein

**Figure 1.**
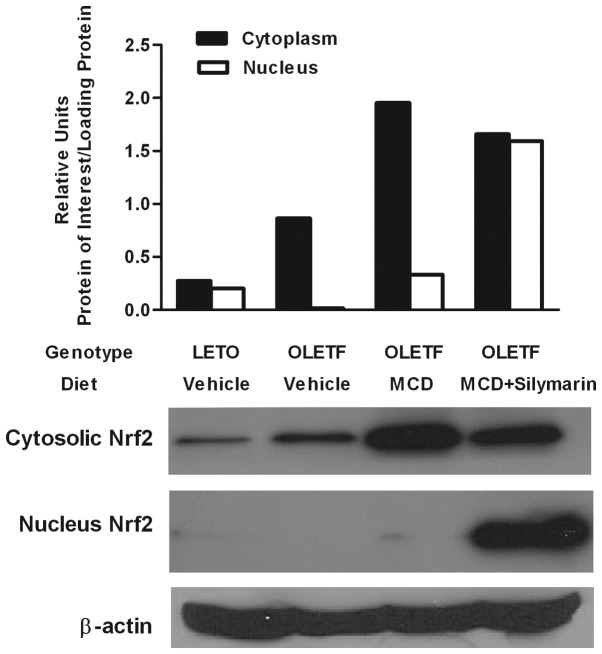
Cytosolic and nuclear Nrf2 protein expression in the liver. Cytosolic Nrf2 protein increased in the livers of MCD diet alone (OLETF/MCD) and MCD with silymarin fed (OLETF/MCD+silymarin) groups. However, only rats in OLETF/MCD+silymarin group had increased nuclear Nrf2.

The liver-protective effect of silymarin has been attributed to its role as an antioxidant (30,31). Since Nrf2 is known to be crucial for several antioxidant responsive elements, resulting in relief of the oxidative burden of cells (32,33), nuclear translocation of Nrf2 was evaluated by western blotting of the cytoplasmic and nuclear protein. Cytoplasmic Nrf2 protein was increased in both MCD diet fed rats with or without silymarin. However, nuclear Nrf2 protein was markedly increased only in the livers of the OLETF/MCD+silymarin group (Fig. 1).

### Silymarin attenuates NAS in the animals MCD-induced NASH model

Histological analysis of NAFLD was performed as suggested by other studies (19–22). Fatty change, inflammation, and existence of hepatocyte ballooning degeneration was assessed and scored separately. Individual scores were added to produce the overall NAS. Feeding MCD diet to OLETF rats resulted in probable or definite NASH as expected. Concomitant administration of silymarin lowered NAS (Table II).

### Effects of silymarin on HSCs in MCD fed insulin-resistant rats

**Figure 2.**
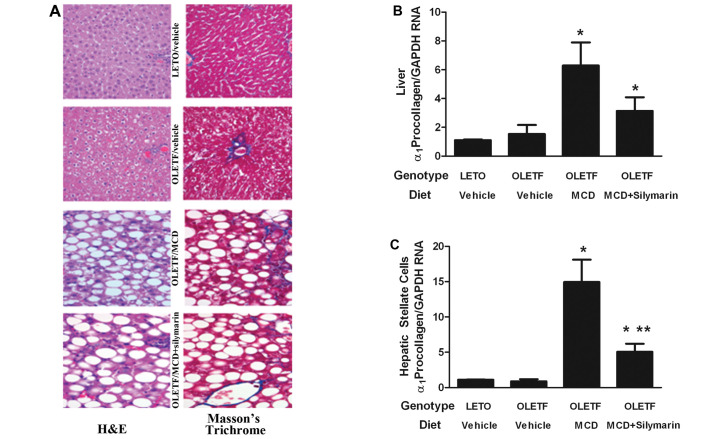
Evaluation of liver fibrosis. (A) Histological analysis after Masson’s trichrome stain showed pericellular fibrosis in MCD fed OLETF rats (OLETF/MCD). Adding silymarin (OLETF/MCD+silymarin) could not completely block MCD induced fibrosis. (B) The OLETF/MCD group had increased α_1_-procollagen mRNA expression in the liver whereas that of rats in OLETF/MCD+silymarin tended to have reduced α_1_-procollagen mRNA. (C) Analysis of hepatic stellate cells (HSCs) isolated from OLETF/MCD rats demonstrated increased α_1_-procollagen mRNA expression whereas HSCs from OLETF/MCD+silymarin had diminished α_1_-procollagen mRNA expression compared to that of OLETF/MCD. ^*^P<0.05 compared to rats without diabetes fed with standard chow (LETO-vehicle). ^**^P<0.05 compared to OLETF/MCD.

Histological evaluation of liver fibrosis was performed after H&E and Masson’s trichrome stain. Despite the significant fatty changes, insulin-resistant OLETF rats with ordinary diet did not demonstrate liver fibrosis. The MCD diet induced mild liver fibrosis confined to fibrosis stage 1. Concomitant administration of silymarin with MCD diet could not completely block the appearance of liver fibrosis (Fig. 2A). Effect of silymarin on expression of α_1_-procollagen mRNA was assessed in the whole liver.

In accordance with the histological analysis, α_1_-procollagen mRNA expression was not enhanced in OLETF rats without MCD diet when compared with LETO rats. Administration of the MCD diet significantly increased the expression of α_1_-procollagen mRNA in the whole liver lysates, which decreased after silymarin administration (Fig. 2B).

**Figure 3.**
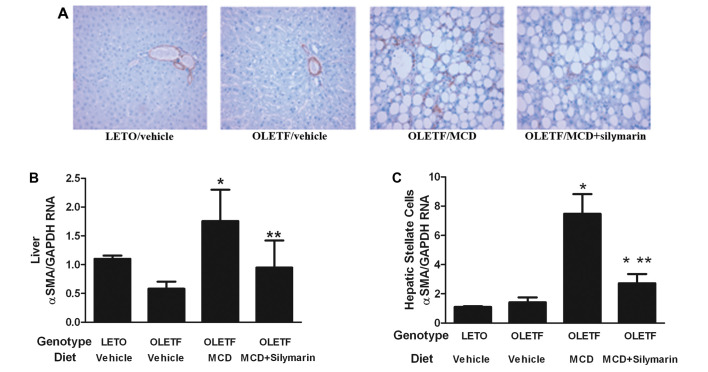
Evaluation of hepatic stellate cell (HSC) activation. (A) Immunohistochemical evaluation showed increased α-SMA positive cells in the liver of OLETF/MCD. The number decreased in OLETF/MCD+silymarin rats. Data are presented as the number of α-SMA positive cells in 30 40x fields (1.3 mm^2^, approximately 3,000 hepatocytes). (B) Analysis of α-SMA mRNA expression in the liver; the results are compatible to these of the immunohistochemical study. (C) Analysis of α-SMA mRNA expression in HSCs, isolated from the experimental animals; the results are in accordance with those of the whole liver study. ^*^P<0.05 compared to LETO/vehicle. ^**^P<0.05 compared to OLETF/MCD.

HSC activation was evaluated in the liver by immunohistochemical analysis and assessment of α-SMA mRNA expression in the whole liver lysates. Feeding the OLETF rats with the MCD diet increased α-SMA positive cells in the liver and concomitant administration of silymarin with the MCD diet significantly decreased these cells (Fig. 3A). There was no significant difference in α-SMA positive cells between LETO and OLETF rats fed with standard diet. Expression of α-SMA mRNA in the liver lysates correlated with the result of the immunohistochemical study (Fig. 3B).

In order to investigate the direct effect of silymarin on HSCs of MCD fed OLETF rats, HSCs were isolated from the experimental animal. Expression of α-SMA mRNA on HSCs from OLETF rats without the MCD diet was compatible with that of the LETO rats. HSCs from MCD fed OLETF rats showed increased α-SMA mRNA levels which were significantly alleviated by adding silymarin (Fig. 3C). This result correlated with the data obtained from the evaluation of the whole liver α-SMA positive cells and liver lysates.

Expression of α_1_-procollagen mRNA was increased in HSCs from MCD fed OLETF rats while no such change was noticed in both OLETF rats without MCD diet and LETO rats. Giving silymarin with the MCD diet significantly relieved this increase in α_1_-procollagen mRNA expression on HSCs (Fig. 2C).

### Association of the effects of silymarin with reduced TNF-α expression on the liver and diminished ERK activation in HSCs

**Figure 4.**
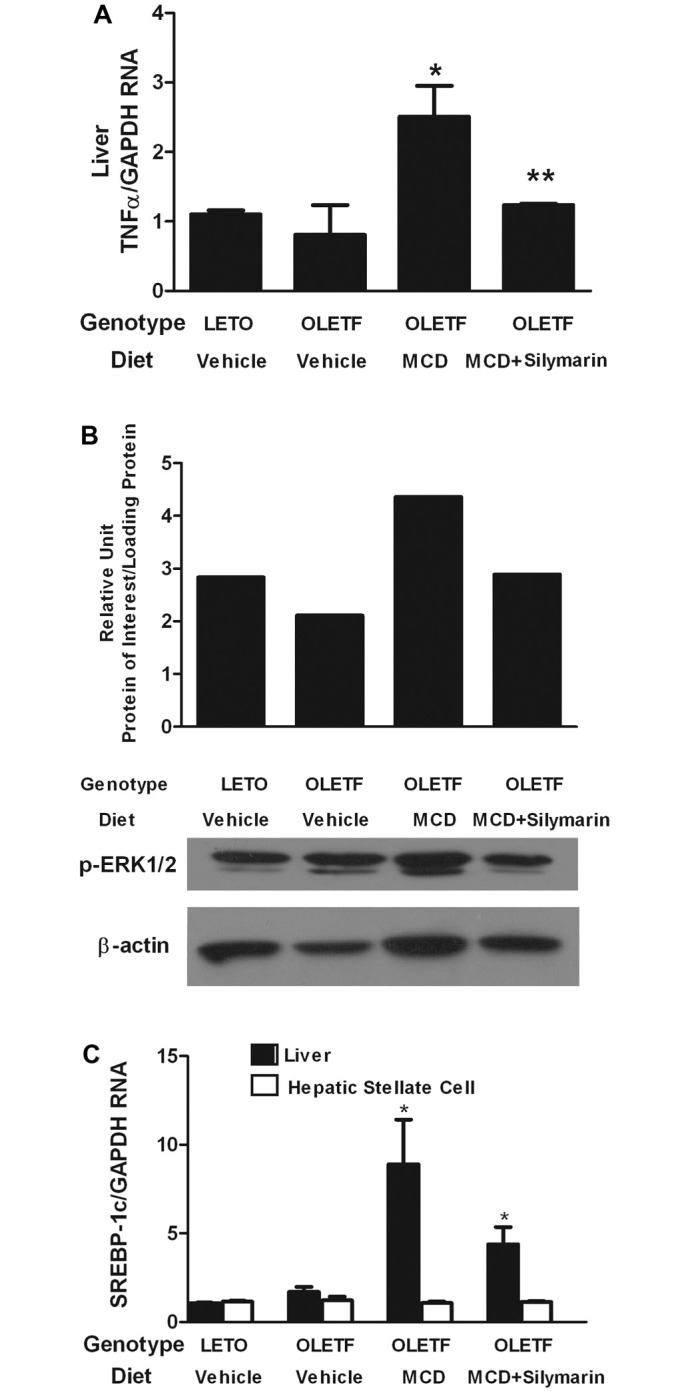
Changes in factors related to non-alcoholic steatohepatitis (NASH). (A) OLETF/vehicle rats display TNF-α mRNA expression compatible to that of LETO/vehicle. OLETF/MCD rats show increased TNF-α mRNA expression, whereas OLETF/MCD+silymarin rats have significantly reduced TNF-α mRNA expression. (B) Activation of extracellular signal-related protein kinase (ERK) was evaluated by detecting phophorylated ERK1/2 protein in HSCs isolated from the experimental animals. (C) Expression of sterol regulatory element-binding protein 1c (SREBP-1c) mRNA increased in the liver of OLETF/MCD rats. Adding silymarin failed to reduce SREBP-1c mRNA expression (OLETF/MCD+silymarin). ^*^P<0.05 compared to LETO/vehicle. ^**^P<0.05 compared to OLETF/MCD.

The role of silymarin on inflammatory cytokine TNF-α was investigated. Expression of TNF-α mRNA on the whole liver of MCD diet fed OLETF rats significantly increased and was further aggravated by the administration of the MCD diet. When silymarin was administered with the MCD diet, expression of TNF-α mRNA was markedly reduced in the liver (Fig. 4A). We examined whether a decrease in TNF-α expression in the liver was accompanied by reduced ERK activation in HSCs. Phosphorylated ERK1/2 protein expression was increased in HSCs isolated from MCD-fed OLETF rats. The effect was reversed by concomitant feeding of silymarin with the MCD diet (Fig. 4B).

The effect of silymarin on SREBP-1c mRNA expression, a transcriptional factor central to the regulation of lipid metabolism, was assessed in the whole liver. Expression of SREBP-1c mRNA was increased in the liver of OLETF rats compared to that of the LETO. Feeding with the MCD diet further increased the expression of SREBP-1c mRNA compared to that of OLETF rats with normal diet. However, concomitant administration of silymarin with the MCD diet could not reduce the expression of SREBP-1c on the transcriptional level in the whole liver tissue (Fig. 4C). There was no significant difference in SREBP-1c mRNA expression on HSCs among four experimental groups.

## Discussion

Concomitant administration of silymarin and the MCD diet to insulin-resistant rats ameliorated the NASH activity score. This result coincided with the suppression of HSC activation and production of α_1_-procollagen. However, this anti-fibrogenic effect of silymarin was not prominent enough so as to be demonstrated under histological analysis. This might be attributed to our failure to generate moderate to severe fibrosis in this particular study. Although several investigations reported that MCD alone would induce NASH with liver fibrosis (34), our preliminary studies all failed to generate fatty liver with considerable inflammation and fibrosis when MCD diet alone was given to wild-type rats. When the MCD diet was fed to rats with systemic insulin resistance, it induced more severe inflammation as previously reported, but it still did not generate fibrosis with more advanced grades.

When the evaluation regarding liver fibrosis was performed in terms of HSC activation, both an *in vivo* and *ex vivo* study showed a significant decrease in activation of HSCs when silymarin was fed along with the MCD diet, thereby supporting the antifibrogenic role of silymarin in diet-induced NASH. We previously reported that HSCs could be isolated from the injured liver, and these cells provided some interesting information on HSC function *in vivo* (23). In the current study, HSC activation was estimated in the liver by counting α-SMA positive cells and by quantification of α-SMA mRNA expression in the whole liver lysates. The results using these two methods were identical, and they were in accordance with the analysis of HSCs isolated from the experimental animal groups.

Apart from the role of silymarin on liver fibrosis, it also seemed to diminish liver inflammation in NASH. Histological analysis showed attenuated inflammation when silymarin was administered along with the MCD diet, and the expression of TNF-α mRNA was also decreased in the liver lysates. It has been reported that TNF-α treated cultured HSCs demonstrated increased proliferation and this effect was associated with ERK activation (34). In accordance with this report, our study showed that isolated HSCs from MCD diet fed OLETF rats had increased expression of p-ERK1/2 protein and this was reversed in rats fed with silymarin and MCD diet.

Nrf2 may upregulate many antioxidant genes. It may play an important role in the adaptive response against oxidative stress (35). Nrf2 activity was reported to increase in cells exposed to oxidative stress, and upon activation, it would translocate into the nucleus (36). In this study, there was increased expression of Nrf2 protein in both MCD fed OLETF rats with and without silymarin administration. However, there seemed to be more effective nuclear translocation of Nrf2 protein in silymarin-fed animals which might lead to the enhanced protective effect against oxidative stress that would accompany NASH.

Our study demonstrates the possible protective effect of silymarin against diet induced NASH by disturbing the role of the inflammatory cytokine, TNF-α, and suppressing the activation of HSCs. Although we used the NASH animal model which is accompanied by systemic insulin resistance so as to simulate human NASH, there are still existing undeniable gaps between the animal model and the actual human disease. Liver fibrosis that is frequently demonstrated in advanced NASH was not prominent in our study. The effect of silymarin on NASH-associated liver fibrosis should be further verified using various animal models.

## Figures and Tables

**Table I.** Changes in body and liver weight.

Characteristic	LETO/vehicle LV	OLETF/vehicle OV	OLETF/MCD OM	OLETF/MCD+silymarin OMS
Initial body weight (g)	530.23±13.79	633.58±30.24^a^	595.95±33.82^a^	577.29±72.70^a^
Final body weight (g)	527.75±16.28	588.00±27.44^a^	411.57±37.17^a,b^	427.80±31.64^a,b^
Liver/final body weight (%)	3.67±0.10	3.73±0.20	4.53±0.16^a,b^	4.30±0.26^a,b^

Otsuka Long-Evans Tokushima Fatty (OLETF) rats, which have been established as animal model of obese type 2 diabetes, were fed with normal chow (vehicle) or methionine and choline-deficient (MCD) diet with or without silymarin for 8 weeks. Silymarin was mixed into the diet (0.5%, weight/weight). Otsuka Long-Evans Tokushima Otsuka (LETO) rats, which originated from the same colony as OLETF rats without diabetes, were fed with normal chow (vehicle) and served as control. Data are expressed as mean ± SD.

a P<0.05 as compared with LETO/vehicle;

b P<0.05, compared with OLETF/vehicle.

**Table II.** Non-alcoholic fatty liver disease (NAFLD) activity score (NAS) of each experimental group assessed 8 weeks after the feeding with experimental diets.

Characteristic	LETO/vehicle LV	OLETF/vehicle OV	OLETF/MCD OM	OLETF/MCD+Silymarin OMS
Steatosis	0.00±0.00	0.75±0.50	3.00±0.00	3.00±0.0
Inflammation	0.00±0.00	0.00±0.00	2.13±0.64	1.80±0.83
Ballooning	0.00±0.00	0.00±0.00	1.88±0.35	1.80±0.44
NAS	0.00±0.00	0.75±0.50	7.00±0.76^a,b^	6.00±0.70^a,b^

Pathological analysis of NAFLD and NAS was performed according to the suggestion by Kleiner *et al* (21). Data are expressed as mean ± SD.

a P<0.05 as compared with LETO/vehicle;

b P<0.05 for the OLETF/MCD vs. the OLETF/MCD+silymarin group.
